# Supplementary material for: Mitochondrial Dysfunction and the Glycolytic Switch Induced by Caveolin-1 Phosphorylation Promote Cancer Cell Migration, Invasion, and Metastasis
Source: Cancers (Basel). 2022 Jun 10;14(12):2862. doi: 10.3390/cancers14122862 (PMC9221213; doi:10.3390/cancers14122862)
Supplement: Supplementary file 1 [file cancers-14-02862-s001.zip › cancers-1700103-supplementary.pdf]

**Supplementary Figure S1:** PTP1B co-immunoprecipitates with CAV1.

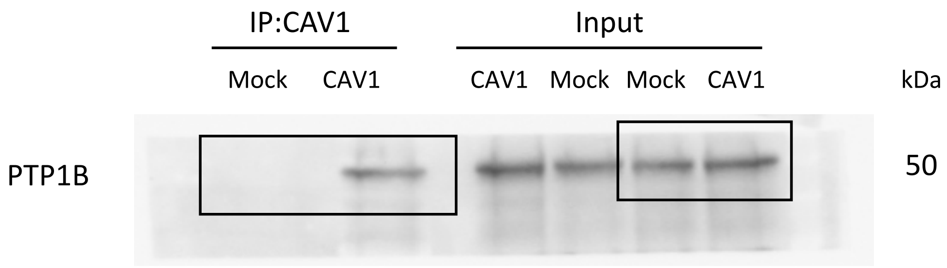


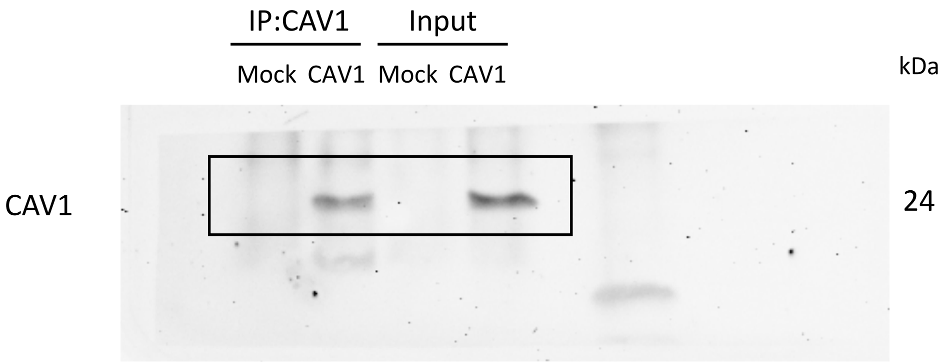


**Densitometry**

| | IP: CAV1 | | Input | |
|---|---|---|---|---|
| Samples | PTP1B | CAV1 | PTP1B | CAV1 |
| Mock | 14235933,000 | 9708154,000 | 7521790,000 | 2229648,000 |
| CAV1 | 31897936,000 | 35365643,000 | 10136326,000 | 41412522,000 |

**Supplementary Figure S2:** Inhibition of PTP1B increases the phosphorylation of CAV1 on tyrosine 14.

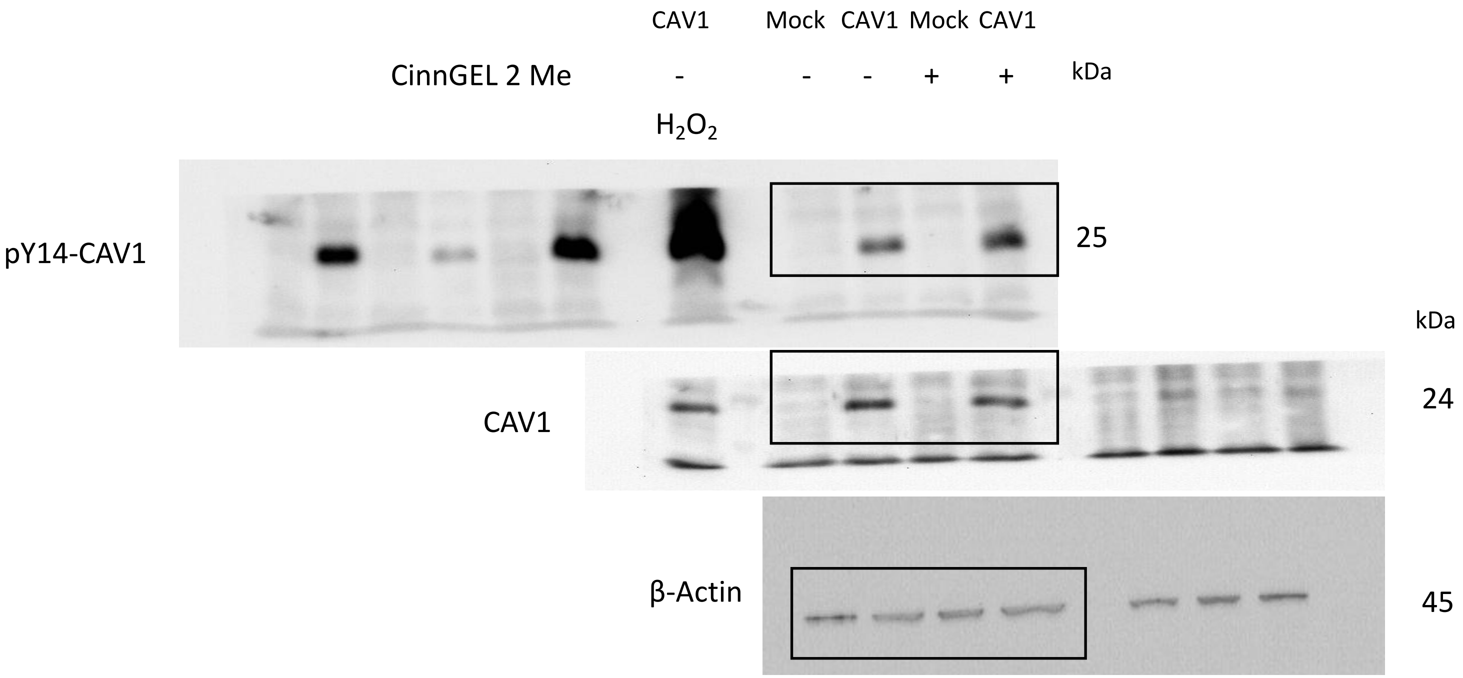


**Densitometry**

| Samples | p-CAV1 | CAV1 | Actin | p-CAV1/CAV1 |
|---|---|---|---|---|
| Mock | 1081,435 | 1088,284 | 4.655.305 | |
| CAV1 | 13998,024 | 16341,125 | 3.664.305 | 0,856613238 |
| Mock CinnGel | 581,728 | 2887,406 | 3.968.012 | |
| CAV1 CinnGel | 18320,589 | 14388,640 | 4.106.669 | 1,273267592 |
